# Chest Wall Resection and Reconstruction Following Cancer

**DOI:** 10.3390/curroncol32120708

**Published:** 2025-12-16

**Authors:** Francesco Petrella, Andrea Cara, Enrico Mario Cassina, Lidia Libretti, Emanuele Pirondini, Federico Raveglia, Maria Chiara Sibilia, Antonio Tuoro

**Affiliations:** 1 Department of Thoracic Surgery, Fondazione IRCCS San Gerardo dei Tintori, 20900 Monza, Italy; andrea.cara@irccs-sangerardo.it (A.C.); enricomario.cassina@irccs-sangerardo.it (E.M.C.); lidia.libretti@irccs-sangerardo.it (L.L.); emanuele.pirondini@irccs-sangerardo.it (E.P.); federico.raveglia@irccs-sangerardo.it (F.R.); maria.sibilia@unimi.it (M.C.S.); antonio.tuoro@irccs-sangerardo.it (A.T.); 2 Department of Oncology and Hemato-Oncology, University of Milan, 20122 Milan, Italy

**Keywords:** chest wall tumors, surgical resection, thoracic oncology, chest wall reconstruction, prosthetic materials, multidisciplinary management

## Abstract

The chest wall represents a complex musculoskeletal structure that provides protection to intrathoracic organs, mechanical support for respiration, and mobility for the upper limbs. Neoplastic diseases of the chest wall encompass a heterogeneous group of benign and malignant lesions, which may be classified as primary—originating from bone, cartilage, muscle, or soft tissue—or secondary, resulting from direct invasion or metastatic spread, most commonly from breast or lung carcinomas. Approximately half of all chest wall tumors are malignant, and their management remains a significant diagnostic and therapeutic challenge. Surgical resection continues to represent the mainstay of curative treatment, with complete en bloc excision and adequate oncologic margins being critical to minimize local recurrence. Advances in reconstructive techniques, including the use of prosthetic materials, biological meshes, and myocutaneous flaps, have markedly improved postoperative stability, respiratory function, and aesthetic outcomes. Optimal management requires a multidisciplinary approach involving thoracic and plastic surgeons, oncologists, and radiotherapists to ensure individualized and comprehensive care. This review summarizes current evidence on the classification, diagnostic evaluation, surgical strategies, and reconstructive options for chest wall tumors, emphasizing recent innovations that have contributed to improved long-term survival and quality of life in affected patients.

## Simple Summary

The chest wall is a complex musculoskeletal structure that provides mechanical support, enables upper limb movement, protects intrathoracic organs, and contributes to respiration. Tumors of the chest wall may be benign or malignant and are classified as primary—arising from bones, cartilage, muscles, or soft tissues—or secondary, resulting from local invasion or metastasis, most often from breast or lung cancer. Approximately half of all chest wall tumors are malignant. Surgical resection remains the cornerstone of treatment and offers the best chance of cure, even when ribs, sternum, or spine are involved. Complete en bloc excision with adequate margins is essential to prevent recurrence, and reconstruction may be required to restore chest stability and respiratory function. Effective management relies on a multidisciplinary approach, integrating advances in surgery, prosthetic materials, and perioperative care to improve outcomes and long-term survival.

## 1. Introduction

The chest wall, or thoracic wall, is a complex musculoskeletal structure that provides mechanical support, facilitates movement of the shoulders and upper limbs, and protects the vital organs housed within the thoracic cavity. It also plays a critical role in normal respiratory mechanics. A variety of benign and malignant lesions can develop within the chest wall, which represent a heterogeneous group of tumors broadly classified as primary or secondary.

Primary chest wall tumors arise from the intrinsic components of the rib cage, including bone, cartilage, muscles, connective tissue, nerves, and blood vessels. Secondary tumors, by contrast, are more common and typically result from local invasion or distant metastases, most frequently from breast or lung carcinoma. Overall, approximately half of all chest wall tumors are malignant in nature [1,2].

The diagnosis and management of these lesions remain among the most challenging tasks in thoracic surgery. Nevertheless, significant progress in recent decades—particularly in surgical techniques and reconstructive materials—has led to improved prognosis and long-term survival. Whenever feasible, complete surgical resection remains the treatment of choice and offers the highest chance of cure. The involvement of the ribs, sternum, or spine should not automatically preclude surgery. En bloc excision with adequate margins of healthy tissue is essential to minimize the risk of local recurrence.

Following resection, chest wall reconstruction is often required to restore anatomical continuity and mechanical stability, as well as to prevent complications such as paradoxical chest motion, respiratory insufficiency, and infection [3]. Optimal management of these complex cases necessitates a multidisciplinary approach, involving thoracic and plastic surgeons, oncologists, radiotherapists, anesthesiologists, and physiotherapists.

The management of chest wall tumors continues to represent a demanding and intricate field of oncologic surgery. This review summarizes the current evidence on these entities, with a focus on contemporary surgical strategies and advances in chest wall resection and reconstruction techniques.

## 2. Primary Chest Wall Tumors

Primary tumors of the chest wall are uncommon and comprise a diverse group of neoplasms, including both benign and malignant types. They account for less than 2% of all thoracic cancers, with malignancies representing approximately 50% to 80% of cases. These tumors are typically classified according to their tissue of origin: about 55% arise from bone or cartilage, while the remaining 45% originate from soft tissue. Among primary bone tumors of the chest wall, roughly 85% develop in the ribs, with 88% of these being malignant. The sternum is affected in about 15% of cases, and tumors in this location are almost always malignant. Notably, over 20% of primary chest wall cancers are discovered incidentally during imaging performed for unrelated reasons [4,5,6].

### 2.1. Primary Bone Tumors

Malignant bone tumors of the chest wall generally have a poor prognosis, with an average five-year survival rate of approximately 60%. Unlike benign lesions, malignant tumors typically exhibit faster growth. The most common clinical symptom is pain, which may or may not be accompanied by a palpable mass. This pain is often mistaken for chronic musculoskeletal discomfort or trauma-related injury. In roughly 20% of cases, chest wall bone tumors are discovered incidentally during radiographic examinations performed for unrelated reasons. Clinical presentations can vary widely, making it challenging to differentiate between malignant and benign tumors based solely on symptoms. For instance, conditions such as Ewing’s sarcoma or Langerhans Cell Histiocytosis may present with nonspecific signs, including generalized fatigue, bone pain, fever, and weakness. A suspicion of malignancy should arise when a mass is detected in a child’s chest wall or when a sternal mass is found in an adult. The presence of a firm, immobile mass adherent to the chest wall increases the likelihood of a bone tumor. Therefore, a comprehensive medical history is essential in the diagnostic workup. Evaluation should include prior cancer history, past radiation exposure, and any history of chest trauma. A meticulous physical examination is critical to confirm the presence, size, and location of the tumor and to guide subsequent diagnostic or surgical planning [7,8].

### 2.2. Primary Soft-Tissue Tumors

Soft-tissue tumors represent about 45% of chest wall malignancies, with soft-tissue sarcomas being the most common malignant type. These tumors most often occur in middle-aged men, though rhabdomyosarcoma is more frequently seen in children. Primary soft-tissue tumors usually present as slow-growing, palpable masses on the chest wall. Rapid growth and associated pain often indicate malignancy, with potential involvement of adjacent structures. However, there are no definitive clinical signs that reliably distinguish malignant from benign tumors. Initial evaluation should include a detailed medical history, assessing for previous malignancies, prior radiation exposure, and the specific symptoms related to the mass [5].

## 3. Secondary Chest Wall Tumors

The chest wall can also be affected by metastatic lesions, which may occur through direct invasion from adjacent structures or via hematogenous and lymphatic spread. Secondary tumors of the thoracic wall are more common than primary chest wall tumors, with metastases from breast and lung cancers being the most frequently observed. In cases of secondary tumors, surgical intervention, including chest wall or sternal resection, is often considered for palliative purposes. The primary goals of such procedures are to alleviate pain, manage ulceration, and control potential complications such as bleeding or infections caused by the tumor mass. When planning surgical resection and possible reconstruction of the chest wall, it is crucial for surgeons to take into account any previous treatments, including chemotherapy, radiotherapy, or prior surgeries performed for the management of the primary tumor. This careful consideration helps optimize outcomes and minimize complications [2,3,4].

### 3.1. Breast Cancer

Advanced breast cancer may extend to the chest wall through direct invasion from the primary tumor, locoregional recurrence in areas previously treated with surgery or radiotherapy, or via hematogenous spread. The sternum can also be affected due to involvement of the internal mammary chain lymph nodes. Patients with chest wall involvement often experience significant symptoms, including pain, compression, ulceration, fungation, bleeding, and bacterial contamination accompanied by malodorous discharge. Treatment strategies for breast cancer are diverse and can include hormonal therapy, systemic chemotherapy, targeted therapies such as monoclonal antibodies, antibody-drug conjugates, or immunotherapy, radiotherapy, surgical intervention, or a combination of these modalities. Decisions regarding chest wall resection and the approach to reconstruct the resulting defect must be highly individualized and typically involve a multidisciplinary team. For patients without distant metastases but with involvement of the musculoskeletal components of the chest wall, local chest wall recurrences, or isolated regional lymph node recurrence, surgical management may offer the best chance for disease control and improved quality of life. Surgical principles focus on achieving complete en bloc resection of the tumor, including all affected or compromised skin, muscle, ribs, and, when necessary, portions of the sternum or clavicles to ensure wide negative margins. Reconstruction of the chest wall defect should prioritize both functional and protective outcomes, safeguarding intrathoracic organs while providing durable coverage, often utilizing various myocutaneous flaps [9,10,11].

### 3.2. Lung Cancer

Chest wall invasion is an uncommon clinical scenario, representing approximately 3% to 8% of all resected non-small-cell lung cancer (NSCLC) cases and around 45% of T3 tumors. Despite its rarity, the optimal management of these advanced tumors remains debated, particularly regarding the roles of neoadjuvant therapy, surgical techniques, and chest wall reconstruction. According to the 2023 National Comprehensive Cancer Network (NCCN) guidelines, en bloc resection is recommended as the primary treatment approach for patients with T3N0–1 disease or resectable T4N0–1 tumors involving the chest wall [12]. In patients without significant functional limitations, there are no absolute contraindications to chest wall resection and reconstruction. For IIIA tumors, surgery should be reconsidered after planned preoperative systemic therapy—either concurrent chemoradiotherapy or chemotherapy—followed by evaluation of postoperative outcomes. Subsequent management, including repeat surgery or additional chemotherapy/chemoradiotherapy, should be guided by the status of surgical margins. For cases with positive margins, reoperation in combination with adjuvant chemotherapy or chemoradiotherapy is generally advised to improve local control and overall outcomes [13,14,15,16].

### 3.3. Others

Isolated metastases to the chest wall originating from extra-thoracic solid tumors are uncommon. Among the malignancies most frequently associated with this pattern of spread are renal cell carcinoma, bladder cancer, prostate cancer, melanoma, thyroid cancer, and ovarian cancer. Although rare, these metastatic lesions can present diagnostic and therapeutic challenges, as they may mimic primary chest wall tumors or other local pathologies. Recognition of the primary tumor type is essential for guiding appropriate management, which may include systemic therapy, surgical excision, or palliative interventions depending on the patient’s overall condition and disease burden [17] [Table 1].

## 4. Diagnostic Approach

### 4.1. Imaging

**Figure 1:**
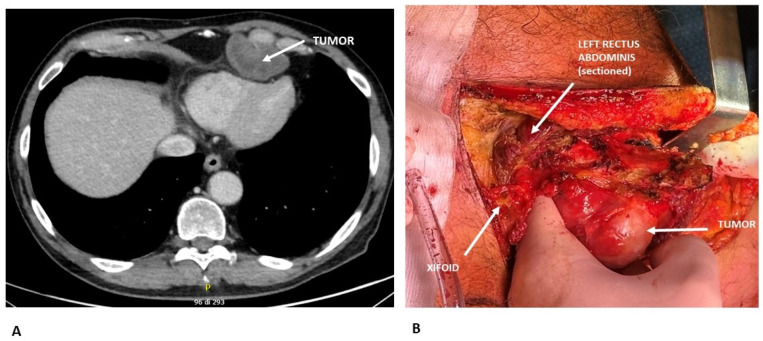
(**A**) Preoperative CT scan showing a chondrosarcoma arising from the 6th costo-chondral junction and compressing the cardiac surface. Radiological features suggesting chondrosarcoma: lobulated, expansive bone lesion with well-defined margins; internal low attenuation due to high water content of the cartilaginous matrix; soft tissue extension beyond the bone. (**B**) Intraoperative view demonstrating surgical resection of the chondrosarcoma.

Radiological diagnosis of chest wall tumors can be challenging due to their considerable histological diversity. Moreover, imaging alone often cannot reliably differentiate between benign and malignant lesions. Therefore, an integrated approach using multiple imaging modalities is recommended. The most commonly employed techniques include chest X-rays, computed tomography (CT), magnetic resonance imaging (MRI), and positron emission tomography (PET). Chest X-rays are typically the first imaging modality used. They help identify the location and size of the lesion and may reveal features such as calcification, bone erosion, or destruction. Certain bone tumors of the chest wall display characteristic radiographic patterns that can facilitate diagnosis; however, early-stage tumors may be overlooked. In some cases, conventional radiography can provide sufficient information for diagnosis, potentially obviating the need for a tissue biopsy [18]. CT imaging offers higher sensitivity and specificity than chest X-rays, providing superior resolution. When performed with contrast, CT enables detailed assessment of the tumor’s extent and its relationship with surrounding structures, including the lungs, pleura, mediastinum, and regional lymph nodes. Additionally, CT can offer insights into tumor density, vascularity, and internal composition [18] [Figure 1]. MRI is particularly valuable for precise tissue characterization and superior spatial resolution. It is especially useful in distinguishing chest wall tumors from infectious or inflammatory conditions, enhancing diagnostic accuracy [19,20,21]. PET is a non-invasive modality that allows for the evaluation of disease presence and extent. It is particularly useful in staging and monitoring treatment response, especially for sarcomas. Studies have reported a sensitivity of 88% and a specificity of 92% for detecting high-grade local sarcoma recurrences, underscoring its important role in clinical management [22] [Table 2].

### 4.2. Pathology

In many cases, radiological imaging alone is not sufficient to establish a definitive diagnosis, and histological confirmation is often required. Therefore, tissue biopsy is commonly performed to verify the diagnosis. For lesions larger than 2 cm, obtaining preoperative diagnostic confirmation is generally recommended [23]. Pathological samples can be collected through fine-needle aspiration, incisional biopsy, or excisional biopsy. Fine-needle aspiration is often appropriate when metastatic disease is suspected. Several studies have also shown that core needle biopsy of bone tumors provides accuracy comparable to that of incisional biopsy. However, the limited tissue obtained via fine-needle aspiration may not always be adequate for a conclusive diagnosis [23]. For tumors larger than 5 cm, incisional biopsy is typically performed, with the understanding that the biopsy site should be re-excised during definitive surgical resection. Excisional biopsy may be considered for small lesions (<2 cm), ensuring that wide negative margins of at least 2 cm are achieved. In such cases, excisional biopsy serves both diagnostic and therapeutic purposes [23].

## 5. Non-Surgical Treatment

Non-surgical treatment of chest wall tumors is highly dependent on tumor histology, location, and extent. For malignant chest wall tumors, especially soft-tissue sarcomas, non-surgical modalities include systemic chemotherapy and radiotherapy, which may be used as neoadjuvant or adjuvant therapy to improve local control, reduce tumor size, or address unresectable disease. Neoadjuvant chemotherapy is commonly employed in approximately 25% of cases to downstage tumors prior to surgical consideration, while adjuvant chemotherapy and radiotherapy are used postoperatively in 39% and 29% of cases, respectively, to reduce recurrence risk and improve survival outcomes [24].

For Ewing sarcoma, neoadjuvant therapy consists of multiagent chemotherapy, typically alternating cycles of vincristine, doxorubicin, and cyclophosphamide (VDC) with ifosfamide and etoposide (IE). This approach is used to reduce tumor burden and treat micrometastatic disease prior to local control (surgery and/or radiotherapy). Dose-intensified regimens with interval compression (every 2 weeks) are now standard in the USA, as supported by recent randomized trials, and are associated with improved event-free and overall survival [25].

For osteosarcoma, neoadjuvant chemotherapy is also standard, most commonly with the MAP regimen (high-dose methotrexate, doxorubicin, and cisplatin). This is administered for 10–12 weeks before surgery, allowing for assessment of histologic response and facilitating limb-sparing procedures. Adjuvant chemotherapy is continued postoperatively. Alternative regimens have not shown superiority to MAP in newly diagnosed cases [26].

In contrast, conventional chondrosarcoma is generally resistant to chemotherapy and radiotherapy. The mainstay of treatment is surgical resection with wide margins. Neoadjuvant therapy is not indicated for conventional chondrosarcoma. However, rare subtypes such as mesenchymal chondrosarcoma may benefit from chemotherapy, and mesenchymal or dedifferentiated variants may be considered for adjuvant or neoadjuvant therapy in select cases [27].

For benign chest wall tumors, observation is appropriate for asymptomatic lesions, and biopsy is indicated when radiological features are unclear or surgery is contraindicated [28]. In select cases where surgery is not feasible due to tumor size, location, or patient comorbidities, radiotherapy may be considered for palliation or local control, particularly in metastatic or locally advanced disease. Multidisciplinary evaluation at specialized sarcoma centers is recommended to individualize treatment plans and optimize outcomes, given the rarity and heterogeneity of these tumors [29]. Overall, non-surgical management is tailored to tumor biology and patient factors, with multimodal therapy playing a critical role in cases where surgical resection is not possible or not indicated [22,23,24].

## 6. Surgical Treatment

**Figure 2:**
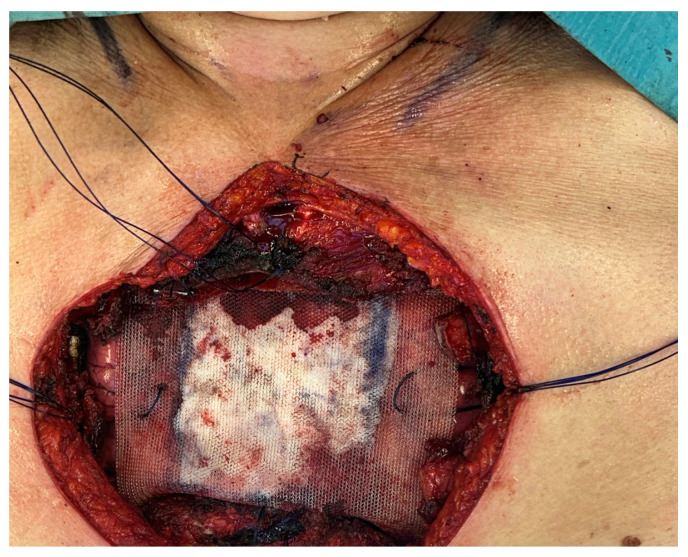
Sternal reconstruction using a composite rigid prosthesis (polypropylene + methyl methacrylate).

Surgical resection remains the cornerstone of treatment for most chest wall tumors, with the principal objective being an “en-bloc” removal of the lesion ensuring microscopically negative margins (R0 resection), as margin status is the key prognostic factor influencing both local recurrence and long-term survival [30,31]. In malignant cases, surgery is typically incorporated into a multidisciplinary strategy that may include neoadjuvant or adjuvant chemotherapy and/or radiotherapy, depending on tumor histology and disease stage. Resections of the chest wall often require complex reconstructive procedures to restore thoracic stability, maintain adequate respiratory function, and protect intrathoracic viscera. Reconstruction options include synthetic meshes, biologic substitutes, and pedicled or free muscle/myocutaneous flaps, selected according to the extent and location of the defect, as well as patient-specific considerations [32]. Close collaboration between thoracic and plastic surgeons is crucial for achieving optimal oncologic clearance and functional recovery. Extended resections involving adjacent structures—such as the lung, diaphragm, or pericardium—can be safely performed in specialized centers without compromising curative potential [31,32]. Ultimately, surgery provides the highest likelihood of cure or durable disease control, with reconstructive techniques continuously advancing to reduce morbidity and improve postoperative quality of life. In cases of metastases, low-grade tumors, or benign lesions, a surgical margin of 2 cm may be adequate. However, for malignant tumors, excision should ensure wide negative margins (R0) tailored to tumor grade and anatomical barriers [33]. For highly malignant tumors, achieving oncological radicality often requires a full-thickness resection, including muscle, bone, and potentially skin [34]. In aggressive tumors that tend to extend along the periosteum, complete removal of the affected bone—whether a rib or the sternum—is necessary, while still preserving respiratory function. Due to the increased likelihood of metastasis to the subperiosteum and surrounding structures, it is generally recommended to resect the ribs immediately above and below the tumor. The tumor should be excised “en bloc,” including the site of any prior biopsy and all involved tissues or structures, such as soft tissue, pleura, lung, or diaphragm. Adequate oncological resection of chest wall tumors may necessitate reconstruction to restore thoracic structural and functional integrity. This helps protect underlying organs, prevents lung herniation and paradoxical chest wall movement, and, when feasible, provides acceptable **aesthetic** outcomes. Currently, there are no definitive guidelines outlining indications for chest wall reconstruction; consequently, surgical approaches often rely on the surgeon’s expertise and preference [35]. Not all chest wall defects require reconstruction. Small defects (<5 cm) or resections involving fewer than three ribs typically do not need prosthetic repair, as soft-tissue coverage is generally sufficient [36]. Subscapular and apico-posterior defects up to 10 cm may also be left unreconstructed due to the stabilizing support of the scapula and shoulder. Several options exist for chest wall reconstruction and stabilization, including soft-tissue flaps and prosthetic materials. Surgeons can choose from synthetic, alloplastic, or biologic prosthetic materials to restore chest wall integrity [36]. The ideal prosthetic material should be rigid enough to prevent paradoxical motion, malleable, resistant to infection, biologically inert, radiolucent, and cost-effective [35]. In practice, no single material meets all these criteria, and combinations are often employed. Each prosthetic option has its advantages and disadvantages, with no clear superiority; the choice of reconstruction method frequently depends on the surgeon’s experience and preference [37] [Figure 2].

Three-dimensional (3D)-printed custom-made implants—including those made from titanium and polyether ether ketone (PEEK)—offer precise anatomical fit and robust mechanical stability. Recent studies demonstrate that 3D-printed PEEK implants, especially when combined with myocutaneous flaps, provide excellent structural support, integration, and functional outcomes with low complication rates in large, full-thickness defects [38].

Titanium plates or bars are widely used for rigid stabilization, particularly in sternal and rib reconstructions. While effective, recent data highlight a high complication rate—including plate fracture, wound healing deficits, and infection—necessitating caution in patient selection and technique [39].

Biosynthetic meshes and acellular dermal matrices (biologic matrices) are primarily used for soft-tissue coverage and semirigid stabilization. Biologic meshes, such as porcine- or ovine-derived matrices, are associated with fewer infections and mesh explantations compared to synthetic meshes, though robust comparative data are lacking [40].

Polyether ether ketone (PEEK), as a 3D-printed implant, is increasingly favored for its biomechanical properties closely resembling cortical bone, with evidence supporting its safety and efficacy in preserving pulmonary function and minimizing complications [38] [Table 3].

## 7. Discussion

Chest wall tumors, encompassing both primary and secondary neoplasms, represent a complex and heterogeneous group of lesions that pose significant diagnostic and therapeutic challenges. Primary tumors, arising from intrinsic chest wall structures such as bone, cartilage, and soft tissue, are rare, constituting less than 2% of thoracic cancers. Despite their low prevalence, primary malignancies account for a substantial proportion of these tumors, with bone and cartilage-derived tumors exhibiting particularly aggressive behavior. Clinical presentation is often nonspecific, with pain and palpable masses being common but not definitive indicators of malignancy. Incidental detection on imaging highlights the importance of thorough diagnostic evaluation, particularly in cases where subtle symptoms may mask underlying pathology. Soft-tissue sarcomas, while less common than bone tumors, demonstrate variable growth patterns and can involve surrounding structures, further complicating early diagnosis [1,2,3,4]. Secondary chest wall tumors, often resulting from metastases or direct invasion by breast or lung carcinomas, are more frequently encountered. In these cases, management is primarily palliative, aiming to relieve pain, prevent ulceration, and maintain quality of life. However, for select patients with localized disease and no distant metastases, surgical intervention can offer meaningful disease control. Advanced breast and lung cancers illustrate the need for individualized treatment strategies that integrate systemic therapies, radiotherapy, and meticulous surgical planning, emphasizing the role of multidisciplinary care [15]. Diagnostic workup for chest wall tumors relies on a combination of imaging modalities and histopathologic confirmation. Chest X-rays, CT, MRI, and PET scans provide complementary information regarding tumor location, extent, and tissue characteristics, facilitating surgical planning and monitoring response to therapy. Biopsy, whether fine-needle, core, or excisional, remains essential to establish histological diagnosis, particularly in lesions larger than 2 cm or those with ambiguous radiological features [21,22,41]. Surgical resection remains the cornerstone of curative treatment, with en bloc excision and negative margins being critical determinants of long-term survival. Reconstruction of the chest wall following extensive resection is often necessary to maintain mechanical stability, protect intrathoracic organs, and preserve respiratory function. Advances in prosthetic materials and flap techniques have improved functional and aesthetic outcomes, although the choice of reconstruction remains highly individualized. For unresectable or metastatic lesions, non-surgical approaches, including chemotherapy, radiotherapy, and targeted therapies, play a vital role in disease management [1,2,42]. Overall, the management of chest wall tumors exemplifies the need for a nuanced, multidisciplinary approach, integrating accurate diagnosis, surgical expertise, and individualized adjunctive therapies to optimize patient outcomes in this challenging oncologic setting.

## 8. Conclusions

Chest wall tumors, whether primary or secondary, represent a rare but clinically significant group of neoplasms requiring careful evaluation and management. Early and accurate diagnosis, integrating imaging and histopathology, is crucial for guiding treatment decisions. Surgical resection with negative margins remains the cornerstone of curative therapy, often necessitating complex reconstruction to preserve thoracic stability and function. Multidisciplinary collaboration is essential, particularly in cases involving metastases or advanced disease, to optimize outcomes through a combination of surgery, systemic therapy, and radiotherapy. Ultimately, individualized, evidence-based management offers the best potential for survival, functional preservation, and improved quality of life.

## Figures and Tables

**Table 1:** Different managements of chest wall tumors.

Tumor Type	Management Approach	Surgical Considerations	Role of Systemic Therapy	Reconstruction Challenges
Primary Sarcoma (e.g., Chondrosarcoma)	Wide en-bloc resection; negative margins essential; MDT involvement	Achieve clear margins; may require extensive resection; reconstruction tailored to defect	Limited efficacy for chemo/radiation except select subtypes	Standard reconstruction; depends on defect size/location
Radiation-Induced Sarcoma	Aggressive surgical resection; aim for negative margins; MDT approach	Poor tissue quality; higher risk of wound complications; negative margins challenging	Limited efficacy; re-irradiation considered in select cases	Increased wound complications; impaired healing; complex reconstruction
Breast Cancer Recurrence	Multimodal: systemic therapy central; salvage surgery if feasible; radiotherapy if not previously given	Salvage mastectomy or repeat conservation; prior treatments impact options	Systemic therapy (chemo, endocrine, anti-HER2) tailored to subtype	Reconstruction affected by prior surgery/radiation; tissue quality variable
NSCLC with T3–T4 Chest Wall Invasion	Induction chemo/immunotherapy followed by surgery in selected N0–N1 cases; MDT evaluation	R0 resection required; complex resection/reconstruction; mediastinal staging essential	Systemic therapy based on molecular profile; surgery not for N2–N3 disease	Extensive defects; may require rigid and soft tissue reconstruction; high morbidity

**Table 2:** Strengths and weaknesses of imaging modalities in chest wall tumors.

Imaging Modality	Strengths	Weaknesses	Specific Clinical Scenarios
CT	Excellent bone detail; detects calcification, cortical destruction, and osseous involvement; rapid acquisition; widely available	Limited soft tissue contrast; may underestimate soft tissue/chest wall invasion; radiation exposure	Initial evaluation, staging, surgical planning, osseous tumors, detection of calcified matrix
MRI	Superior soft tissue contrast; precise delineation of tumor margins and extent; optimal for neurovascular, muscle, and fat involvement; no ionizing radiation	Longer scan time; higher cost; motion artifacts; less effective for calcification	Problem-solving for equivocal CT findings, preoperative planning, assessment of soft tissue and neurovascular invasion
Ultrasound	High sensitivity/specificity for superficial chest wall invasion; real-time imaging; guides biopsy; no radiation	Operator-dependent; limited for deep or complex lesions; poor bone detail	Assessment of chest wall invasion by lung cancer, superficial lesions, biopsy guidance
PET/CT	Functional imaging; detects metabolic activity; useful for staging, treatment response, recurrence	Poor anatomical detail; not first-line for initial evaluation; limited soft tissue characterization	Staging, evaluation of treatment response, detection of recurrence
Chest Radiograph	Readily available; detects mineralization and gross bone changes	Low sensitivity for soft tissue and small lesions; poor for extent and tissue characterization	Initial screening, detection of mineralization in bone tumors

**Table 3:** Advantages and disadvantages of different reconstruction materials.

Material	Advantages	Disadvantages
Synthetic Meshes [40]	Widely available; strong; cost-effective; easy to handle; good for large defects	Higher risk of infection and surgical site complications, especially in irradiated or obese patients; may require explantation if infected; less tissue integration
Biologic Matrices [40]	Lower infection rates; better tissue integration; suitable for contaminated or irradiated fields; fewer mesh explantations	Higher cost; increased bacterial adhesion in vitro; less mechanical strength than synthetic mesh; limited long-term data
Titanium Implants (plates/bars/mesh) [39]	Rigid stabilization; biocompatible; good for large, full-thickness defects; easy to shape and fix; durable	High complication rate (plate fracture, wound healing deficit, infection); may require revision surgery; caution in patient selection
3D-Printed Custom-Made Implants (Titanium, PEEK, Methyl Methacrylate) [38]	Precise anatomical fit; customizable for complex defects; excellent structural support; improved functional and cosmetic outcomes; low complication rates with PEEK	Higher cost; limited long-term data; risk of infection (especially with titanium); technical expertise required

## Data Availability

Data are available on request.

## Abbreviations

The following abbreviations are used in this manuscript:

NSCLC	Non-small-cell Lung Cancer
NCCN	National Comprehensive Cancer Network
CT	Computed Tomography
MRI	Magnetic Resonance Imaging
PET	Positron Emission Tomography

## References

[1] Poulos C.M., Servais E.L. (2025). Precision Medicine in Thoracic Surgery: Chest Wall, Pleural, and Mediastinal Malignancies. Thorac. Surg. Clin..

[2] Oreglio C., Grimaldi C., Gonfiotti A., Fusi G., Severi E., Piccolo R.L., Beltrami G., Tamburini A., Buccoliero A.M., Cianci M.C. (2025). Exploring the Role of Bioprosthesis for Chest Wall Reconstruction in Pediatric Oncology. Pediatr. Blood Cancer.

[3] Petrella F., Spaggiari L. (2020). Surgery of the chest wall: Indications, timing and technical aspects. J. Thorac. Dis..

[4] Gonfiotti A., Salvicchi A., Voltolini L. (2022). Chest-Wall Tumors and Surgical Techniques: State-of-the-Art and Our Institutional Experience. J. Clin. Med..

[5] Cipriano A., Burfeind W. (2017). Management of Primary Soft Tissue Tumors of the Chest Wall. Thorac. Surg. Clin..

[6] Thomas M., Shen K.R. (2017). Primary Tumors of the Osseous Chest Wall and Their Management. Thorac. Surg. Clin..

[7] Shah A.A., D’Amico T.A. (2010). Primary chest wall tumors. J. Am. Coll. Surg..

[8] Sabanathan S., Salama F.D., Morgan W.E., Harvey J.A. (1985). Primary chest wall tumors. Ann. Thorac. Surg..

[9] Baldes N., Grapatsas K., Dörr F., Menghesha H., Schuler M., Welt A., Stuschke M., Kimmig R., Hoffmann O., Bölükbas S. (2024). Chest wall resections for advanced breast cancer: A narrative review. J. Thorac. Dis..

[10] Vagia E., Cristofanilli M. (2021). New Treatment Strategies for the Inflammatory Breast Cancer. Curr. Treat. Options Oncol..

[11] Petrella F., Lo Iacono G., Casiraghi M., Gherzi L., Prisciandaro E., Garusi C., Spaggiari L. (2020). Chest wall resection and reconstruction by composite prosthesis for locally recurrent breast carcinoma. J. Thorac. Dis..

[12] Riely G.J., Wood D.E., Aisner D.L., Loo B.W., Axtell A.L., Bauman J.R., Bharat A., Chang J.Y., Desai A., Dilling T.J. (2025). NCCN Guidelines^®^ Insights: Non-Small Cell Lung Cancer, Version 7.2025. J. Natl. Compr. Canc. Netw..

[13] Huang L., Li F., Neudecker J., Elsner A., Strauchmann J., Dziodzio T., Zhou H., Rueckert J. (2024). Chest wall resections for non-small cell lung cancer: A literature review. J. Thorac. Dis..

[14] Bonis A., Detterbeck F., Figueroa P.U., Chen H., Osarogiagbon R., Dell’Amore A., Infante M. (2025). Classification of recurrence patterns in surgically treated non-small cell lung cancer—A systematic review and a call for standardization. Eur. J. Surg. Oncol..

[15] Wang L., Yan X., Zhao J., Chen C., Chen C., Chen J., Chen K.N., Cao T., Chen M.W., Duan H. (2021). Expert consensus on resection of chest wall tumors and chest wall reconstruction. Transl. Lung Cancer Res..

[16] Rizzo S., Raimondi S., de Jong E.E.C., van Elmpt W., De Piano F., Petrella F., Bagnardi V., Jochems A., Bellomi M., Dingemans A.M. (2019). Genomics of non-small cell lung cancer (NSCLC): Association between CT-based imaging features and EGFR and K-RAS mutations in 122 patients—An external validation. Eur. J. Radiol..

[17] Carsote M., Terzea D., Vasilescu F., Cucu A.P., Ciuche A., Nistor C. (2023). Sternum Metastases: From Case-Identifying Strategy to Multidisciplinary Management. Diagnostics.

[18] Tateishi U., Gladish G.W., Kusumoto M., Hasegawa T., Yokoyama R., Tsuchiya R., Moriyama N. (2003). Chest wall tumors: Radiologic findings and pathologic correlation: Part 1. Benign tumors. Radiographics.

[19] Bueno J., Lichtenberger J.P., Rauch G. (2018). MR Imaging of Primary Chest Wall Neoplasms. Top. Magn. Reson. Imaging.

[20] Genovese E., Canì A., Rizzo S., Angeretti M.G., Leonardi A., Fugazzola C. (2011). Comparison between MRI with spin-echo echo-planar diffusion-weighted sequence (DWI) and histology in the diagnosis of soft-tissue tumours. Radiol. Med..

[21] Chianca V., Albano D., Messina C., Vincenzo G., Rizzo S., Del Grande F., Sconfienza L.M. (2021). An update in musculoskeletal tumors: From quantitative imaging to radiomics. Radiol. Med..

[22] Schwarzbach M.H., Dimitrakopoulou-Strauss A., Willeke F., Hinz U., Strauss L.G., Zhang Y.M., Mechtersheimer G., Attigah N., Lehnert T., Herfarth C. (2000). Clinical value of [18-F] fluorodeoxyglucose positron emission tomography imaging in soft tissue sarcomas. Ann. Surg..

[23] Kiatisevi P., Thanakit V., Sukunthanak B., Boonthatip M., Bumrungchart S., Witoonchart K. (2013). Computed tomography-guided core needle biopsy versus incisional biopsy in diagnosing musculoskeletal lesions. J. Orthop. Surg..

[24] Sharma J., Deo S.V.S., Kumar S., Bhoriwal S., Gupta N., Saikia J., Bhatnagar S., Mishra S., Bharti S., Thulkar S. (2024). Malignant Chest Wall Tumors: Complex Defects and Their Management—A Review of 181 Cases. Ann. Surg. Oncol..

[25] Brennan B., Kirton L., Marec-Bérard P., Gaspar N., Laurence V., Martín-Broto J., Sastre A., Gelderblom H., Owens C., Fenwick N. (2022). Comparison of two chemotherapy regimens in patients with newly diagnosed Ewing sarcoma (EE2012): An open-label, randomised, phase 3 trial. Lancet.

[26] Meltzer P.S., Helman L.J. (2021). New Horizons in the Treatment of Osteosarcoma. N. Engl. J. Med..

[27] Weinschenk R.C., Wang W.L., Lewis V.O. (2021). Chondrosarcoma. J. Am. Acad. Orthop. Surg..

[28] Minervini F., Sergi C.M., Scarci M., Kestenholz P.B., Valentini L., Boschetti L., Bertoglio P. (2024). Benign tumors of the chest wall. J. Thorac. Dis..

[29] van Roozendaal L.M., Bosmans J.W.A.M., Daemen J.H.T., Franssen A.J.P.M., van Bastelaar J., Engelen S.M.E., Keymeulen K.B.M.I., Aguiar W.W.S., de Campos J.R.M., Hulsewé K.W.E. (2024). Management of soft tissue sarcomas of the chest wall: A comprehensive overview. J. Thorac. Dis..

[30] Scarnecchia E., Liparulo V., Capozzi R., Ceccarelli S., Puma F., Vannucci J. (2018). Chest wall resection and reconstruction for tumors: Analysis of oncological and functional outcome. J. Thorac. Dis..

[31] Collaud S., Stork T., Dirksen U., Pöttgen C., Hegedüs B., Schildhaus H.U., Bauer S., Aigner C. (2021). Surgical Treatment for Primary Chest Wall Sarcoma: A Single-Institution Study. J. Surg. Res..

[32] Crowley T.P., Atkinson K., Bayliss C.D., Barnard S., Milner R.H., Ragbir M. (2020). The surgical management of sarcomas of the chest wall: A 13-year single institution experience. J. Plast. Reconstr. Aesthet. Surg..

[33] Lo Iacono G., Mazzella A., Mohamed S., Petrella F., Sedda G., Casiraghi M., Girelli L., Bertolaccini L., Spaggiari L. (2023). The Role of Surgery in Primary Chest Wall Tumors: Over 20 Years’ Experience in Resection and Reconstruction. Cancers.

[34] Hazel K., Weyant M.J. (2015). Chest Wall Resection and Reconstruction: Management of Complications. Thorac. Surg. Clin..

[35] Seder C.W., Rocco G. (2016). Chest wall reconstruction after extended resection. J. Thorac. Dis..

[36] Khullar O.V., Fernandez F.G. (2017). Prosthetic Reconstruction of the Chest Wall. Thorac. Surg. Clin..

[37] Weyant M.J., Bains M.S., Venkatraman E., Downey R.J., Park B.J., Flores R.M., Rizk N., Rusch V.W. (2006). Results of chest wall resection and reconstruction with and without rigid prosthesis. Ann. Thorac. Surg..

[38] Huo J.Y., Li Y.Q., Zhao C.Y., Zhao Z.W., Du J.C., Wang L., Yang S.H., Duan C.Z., Zhao Y., Huang L.J. (2025). Combined latissimus dorsi myocutaneous flap and 3D-printed PEEK implant for reconstruction of a large full-thickness chest wall defect: A retrospective study. J. Plast. Reconstr. Aesthet. Surg..

[39] Bergovec M., Smolle M., Lindenmann J., Fediuk M., Leithner A., Smolle-Jüttner F.M. (2022). High complication rate with titanium plates for chest wall reconstruction following tumour resection. Eur. J. Cardiothorac Surg..

[40] Velotta J.B., Hammer J., Mukhatyar V. (2025). Chest Wall Reconstruction Using Biologic Mesh to Cover Soft Tissue Defects: A Narrative Review. J. Surg. Res..

[41] Fanti S., Farsad M., Battista G., Monetti F., Montini G.C., Chiti A., Savelli G., Petrella F., Bini A., Nanni C. (2003). Somatostatin receptor scintigraphy for bronchial carcinoid follow-up. Clin. Nucl. Med..

[42] Pelosi G., Petrella F., Sandri M.T., Spaggiari L., Galetta D., Viale G. (2006). A primary pure yolk sac tumor of the lung exhibiting CDX-2 immunoreactivity and increased serum levels of alkaline phosphatase intestinal isoenzyme. Int. J. Surg. Pathol..

